# Enhancing nitrogen use efficiency and yield of maize (*Zea mays* L.) through Ammonia volatilization mitigation and nitrogen management approaches

**DOI:** 10.1186/s12870-024-04749-7

**Published:** 2024-01-27

**Authors:** Khadim Dawar, Atif Dawar, Muhammad Tariq, Ishaq Ahmad Mian, Asim Muhammad, Laiba Farid, Shadman Khan, Kashif Khan, Shah Fahad, Subhan Danish, Abdullah Ahmed Al-Ghamdi, Mohamed S. Elshikh, Muhammad Tahzeeb-ul-Hassan

**Affiliations:** 1 Department of Soil and Environmental Science, The University of Agriculture Peshawar, Peshawar, Pakistan; 2 Department of Agronomy, Faculty of Crop Production Sciences, The University of Agriculture Peshawar, Peshawar, Khyber Pakhtunkhwa 25130 Pakistan; 3 Department of Agronomy, Abdul Wali Khan University Mardan, Mardan, Khyber Pakhtunkhwa 23200 Pakistan; 4 Department of Natural Sciences, Lebanese American University, Byblos, Lebanon; 5 Department of Soil Science, Faculty of Agricultural Sciences and Technology, Bahauddin Zakariya University, Multan, Punjab Pakistan; 6 Department of Botany and Microbiology, College of Science, King Saud University, P.O. 2455, Riyadh, 11451 Saudi Arabia; 7 Charles Darwin University, Casurina Campus Darwin NT, Darwin, 0812 Australia

**Keywords:** Ammonia emission, Growth attributes, Maize, N use efficiency (NUE), Sulfur-coated urea, Yield attributes

## Abstract

Management of nitrogen (N) fertilizer is a critical factor that can improve maize (*Zea mays L.*) production. On the other hand, high volatilization losses of N also pollute the air. A field experiment was established using a silt clay soil to examine the effect of sulfur-coated urea and sulfur from gypsum on ammonia (NH_3_) emission, N use efficiency (NUE), and the productivity of maize crop under alkaline calcareous soil. The experimental design was a randomized complete block (RCBD) with seven treatments in three replicates: control with no N, urea_150_ alone (150 kg N ha^−1^), urea_200_ alone (200 kg N ha^−1^), urea_150_ + S (60 kg ha^−1^ S from gypsum), urea_200_ + S, SCU_150_ (sulfur-coated urea) and SCU_200_. The results showed that the urea_150_ + S and urea_200_ + S significantly reduced the total NH_3_ by (58 and 42%) as compared with the sole application urea_200_. The NH_3_ emission reduced further in the treatment with SCU_150_ and SCU_200_ by 74 and 65%, respectively, compared to the treatment with urea_200_. The maize plant biomass, grain yield, and total N uptake enhanced by 5–14%, 4–17%, and 7–13, respectively, in the treatments with urea_150_ + s and urea_200_ + S, relative to the treatment with urea_200_ alone. Biomass, grain yield, and total N uptake further increased significantly by 22–30%, 25–28%, and 26–31%, respectively, in the treatments with SCU_150_ and SCU_200_, relative to the treatment with urea_200_ alone. The applications of SCU_150_ enhanced the nitrogen use efficiency (NUE) by (72%) and SCU_200_ by (62%) respectively, compared with the sole application of urea_200_ alone. In conclusion, applying S-coated urea at a lower rate of 150 kg N ha^−1^ compared with a higher rate of 200 kg N ha^−1^ may be an effective way to reduce N fertilizer application rate and mitigate NH_3_ emission, improve NUE, and increase maize yield. More investigations are suggested under different soil textures and climatic conditions to declare S-coated urea at 150 kg N ha^−1^ as the best application rate for maize to enhance maize growth and yield.

## Introduction

Urea accounts for 50% of the total world N-consumption [1], and its use in Pakistan has also increased sharply. It is the most extensively utilized form of N as fertilizer for Cropping and grasslands [2]. However, urea’s volatile nature results in significant environmental losses as N (30–50%) during plant growth [3]. The losses of N as nitrous oxides (N_2_O) and NH_3_ from the top surface of soil through hydrolysis depended on various chemical and physical properties of the soil such as alkalinity and calcareousness of the soil, its cation and anion exchange capacity, pattern of rainfall, airspeed, soil temperature and rate of humidity and urease activity [4–6]. Furthermore, the amount of ammonium (NH_4_^+^) and NH_3_ are related to the pH of the corresponding soil in relation to the granules of applied fertilizers, which is directly related to NH_3_ volatilization [7, 8].

According to Proctor et al. [9], the alkaline calcareousness nature of the soil leads to the braking of urea through hydrolysis so rapidly, due to which the pH of the soil significantly rises to above 8.2 and the emission rate of nitrogen as ammonia increases at faster rate [10, 11]. The amount of NH_3_-N fertilizer that is volatilized is significantly increased when the pH of the corresponding soil increases from 7 [12]. Urea applied to soil reacts with water through urease and quickly transforms to NH_4_^+^. The soil pH at the reaction site rises because of the consumption of H^+^ ions and the production of NH_4_ and HCO_3_ [13].

Many new management practices and technologies have already been developed to minimize N losses to the atmosphere and optimize N utilization. Management practices such as principles of 4R, such as right dose, right time, right place, and right source, are critical considerations in reducing N losses due to volatilization [14, 15]. Several technologies, such as timely released fertilizers, stable fertilizers, controlled-release fertilizers, and their blends, have already been adopted to reduce N losses and enhance N use efficiency [16–20].

To maximize the reduction of nitrogen losses and bolster nitrogen efficiency, employing various management techniques—such as avoiding heavy N application rates, timing N fertilizer applications appropriately, splitting N applications, and incorporating urease inhibitors into urea—may hold the highest potential [13, 21, 22]. In addition, there are other methods for minimizing NH_3_ loss. One such technology is the use of S coated with urea, which has recently received significant attention to delay urea hydrolysis by lessening urease activity [23, 24], enhancing plant growth, and increasing N use efficiency [16, 25, 26].

However, there are limited published works on the effectiveness of urea coated with S on the losses of NH_3_ and maize yield from cultivated regions of Pakistan under alkaline calcareous soil and hot climatic conditions. The novelty of this study lies in its groundbreaking approach to maize cultivation, merging conventional inorganic fertilization with sustainable organic amendments. This innovative combination addresses the imperative of enhancing nitrogen use efficiency (NUE) and pioneers a holistic strategy for mitigating ammonia volatilization, a frequently underestimated facet of nitrogen loss in agriculture. By synergizing these diverse elements, the research seeks to unlock the untapped potential for elevated maize yields, thereby presenting a novel solution to the pressing global challenges of food security and sustainable agricultural practices. Therefore, this study aimed to investigate the effect of applying S-coated urea with NI alone or in combination with urea+S at different rates on NH_3_ emission, crop productivity, and N use efficiency.

## Material and methods

### Description of the experimental site

**Fig. 1:**
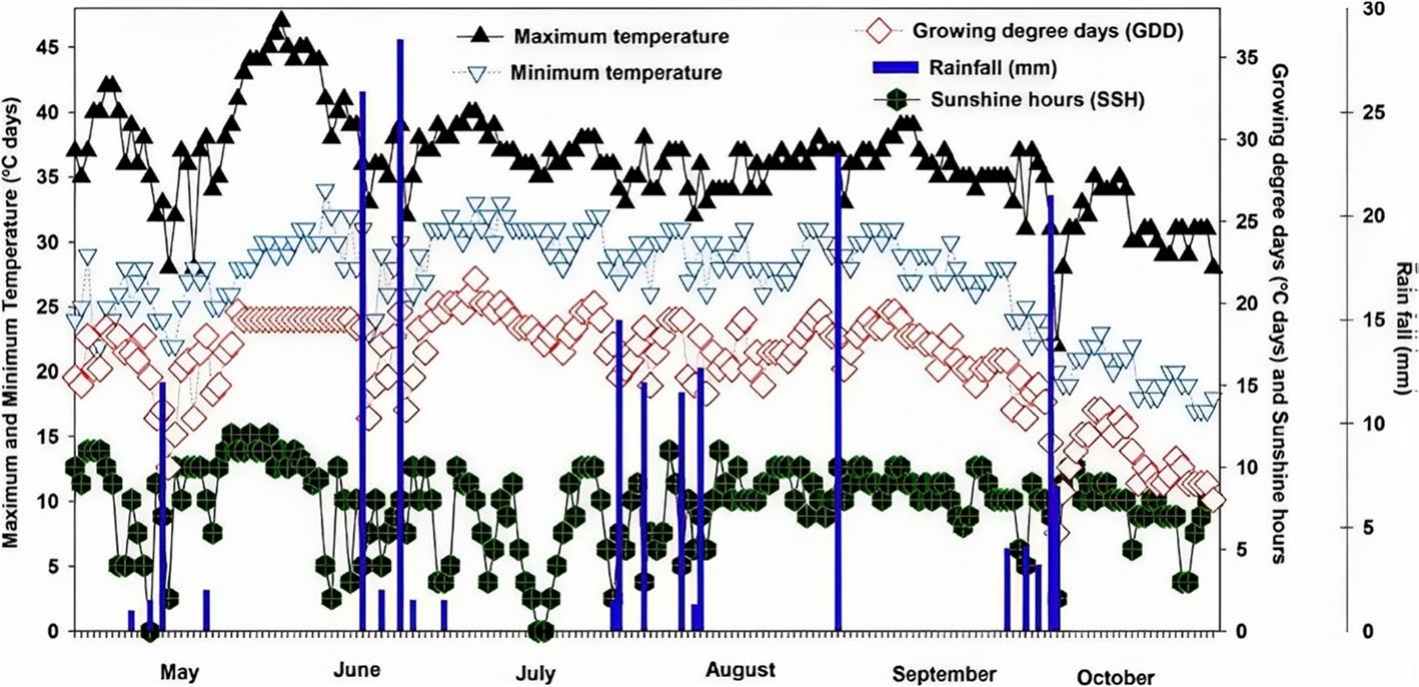
Weather conditions for the 2020 maize growing season, including highest and lowest temperatures, sunlight hours, rising degree days, and rainfall

The field experiment was conducted at a research farm (34.1°′21″ N, 71°28′5′E), at the University of Agriculture Peshawar, Pakistan. The experimental area altitude from sea level was 350 m, had semi-arid climatic and soil conditions, with 383 mm annual rainfall with an average air temperature of 24 °C, while the mean summer and winter temperature also shown in Fig. 1. This arable site has been under an irrigated maize-wheat crop rotation system for over 10 years. According to IUSS Working Group WRB (2006), the soil at the study site was cambisol. The soil was silty clay loam and alkaline calcareous (pH 8.23) with an electrical conductivity (EC) of 0.16 dS m^−1^ (Table 1).

**Table 1:** Physico-chemical soil properties of the experimental site

Characteristics	Unit	Value
Sand	%	30.5
Silt		64.1
Clay		5.4
Textural class		Silt loam
pH (1:5)		8.23
ECe (1:5)	d.Sm^−1^	0.16
Soil organic matter	(%)	0.73
Total Mineral N (NH_4_^+^-N + NO_3_^−^-N)	(mg kg^−1^)	0.56
Available SO_4_-S	(mg kg^−1^)	14.79

### Experimental site

The tillage operation in the corresponding soil was carefully done with mould board plough to a depth of 0.30 m, following the rotavator, before the sowing. The soil was ploughed two times across the field and twice up and down it. The rotovator was used to bury the previous crop residue at 4–6 cm depth. After tillage, planking was done in all plots to break the clods and smooth the field.

### Seeds sowing

Seeds of the maize variety Pioneer 3025 were sown at a rate of 30 kg ha^−1^ on June 24, 2020. The plot was maintained at 5 × 5 m, with a plant-to-plant spacing of 25 to 30 cm and a row-to-row distance of 70 cm.

### Fertilizer

Before sowing of maize crop, all the experimental units were fertilized with the P and K basal dose such as P_2_O_5_ ha^−1^ 90 kg, from source *di-ammonium phosphate* (DAP) and 60 kg K_2_O ha^−1^ from the source and sulfate of potash (SOP), respectively. Nitrogen was surface applied in the form of urea in two split applications, one half during the first irrigation (July 5, 2020) and the other half when the maize plants were at knee height (August 10, 2020).

### Treatment plan

The experimental design was a randomized complete block, consisting of the following seven treatments in three replicates: control with no N, urea_150_ alone (at 150 kg N ha^−1^), urea_200_ alone (at 200 kg N ha^−1^), urea_150_ + S (S 60 kg ha^−1^ from gypsum), urea_200_ + S, sulfur-coated urea (SCU_150_) and SCU_200_. Sulfur-coated urea was prepared manually using a rotating drum [27]. Granular urea measuring 38 kg was taken in a rotating drum. To adhere sulfur coating on urea, acacia gum was used (also known as gum Arabic). It is considered a natural polymer and can be used for agricultural purposes [28]. Keeping the rate of 2 g acacia gum for 1 kg urea, 76 mL of acacia solution was prepared and sprinkled on urea in a drum as suggested by Shivay et al. [29] previously. The drum was rotated for 10 min, then 2 kg sulfur powder (99%) was added to the drum, and the drum was rotated for 15 min. Urea was taken out of the drum and spread out on a plastic sheet under shade for 10 min and stored. This coated urea consisted of 5% (w/w) sulfur.

### Ammonia measurement and analysis

For the measurement of ammonia emissions, the [30] and [31] described procedures were followed, in which a transparent 1.5 L plastic bottle of soft drink was placed on the field with the removal of its bottom after the application of nitrogen fertilizers, the chamber for collecting NH_3_ was mounted at the top of the bottle. The area covered by each bottle is 10 cm in diameter with 78.5 cm^2^. A foam strip that was 2.5 cm by 25 cm and 3 mm thick that had been pre-soaked in acid solution [1 M sulphuric acid (H_2_SO_4_) + 2%(vol/vol) glycerol] was placed inside each chamber along with a polythene jar (60 mL) that held the acidic solution to maintain the foam strip moist throughout the sample times. Each plot received a single NH_3_ chamber. Daily foams were collected during the first 7 and 12 days following the fertilizer treatment. The plastic pots used to transport the collected foam strips to the lab were then properly rinsed with 40 mL of deionized water before being put into flasks. The Erlenmeyer flasks were shaken by a shaker for 20 minutes. After carefully cleaning the foams, the collected samples from the foam were taken in a conical flask and filled to 100 mL with distilled water. After this, the sample was used to determine NH_4_^+^ followed [32] procedure.

The NH_3_ fluxes (kg N ha^−1^ d^−1^) were calculated using the following equation:

$$\textrm{F}=\frac{2\times \textrm{C}\times \textrm{V}\times 14\times {10}^{-2}}{\uppi \times {\textrm{r}}^2}\times \frac{24\ }{\textrm{t}}$$

Where;

C: Concentration of standard sulfuric acid (mol L^−1^).

V: Volume of standard sulfuric acid used in the titration (ml).

T: duration of collection (h).

R: Radius chamber radius (m).

The cumulative NH_3_ emissions were the sum of NH_3_ fluxes on sampling days.

### Soil sampling and analysis

Before any treatments were applied, ten soil cores ranging from 0 to 10 cm in depth were extracted from the experimental site. After removing the visible plant debris and visible roots, the soil was sieved using a 2-mm mesh. Important soil characteristics were investigated during the sieved soil sample examination (Table 1). Using the [33] approach, the pH of the soil was measured in the saturated soil extract. Using an EC meter and a soil water suspension (1:5), the electrical conductivity (EC) in the soil extract was measured in accordance with the protocol [34]. According to Nelson and Sommers [35] description of the Walkley-Black process, the amount of soil organic matter (OM) was measured. The texture of the soil was measured by following [36] procedure. The steam distillation technique determines the mineral N content of soil [32]. This procedure involved mixing a 20 g sample of damp soil with 100 mL of 1 M KCl for an hour before filtering; MgO or MgO with devarday’s alloy was used with the 20 mL of sample in wolf bottle for obtaining NH_4_^+^ or total mineral N. For analysis of sulfur, 50 mL of 0.001 M CaCl_2_.2H_2_O was added to 25 mg of soil to determine the amount of sulfur present. Following 30 minutes of shaking, Whatman filter papers (42 numbers) were used to filter the solution. In a 25 mL flask, 1 mL of the aliquot solution and 5 mL of mixed acidic reagent were added with 1 mL of acidic sulfate. After 3 minutes, 0.5 g of fine BaCl_2_ in 2H_2_O powder was added. After that, a reagent called Acacia was introduced, and SO_4_-S was analyzed using a spectrophotometer [37].

$${\textrm{SO}}_4-\textrm{S}\ \left({\textrm{mg L}}^{-1}\right)=\frac{{\textrm{SO}}_4-\textrm{S}\Big(\textrm{from}\ \textrm{cal}.\;\textrm{curve}\times \textrm{A}\ \left(\textrm{total}\ \textrm{extr}.\;\textrm{vol}\right))}{\textrm{W}.\textrm{t}\ \textrm{of}\ \textrm{sample}}$$

### Plant sampling and analysis

The mature plant samples were collected for nutrient analysis. Following collection, the plant material underwent a meticulous purification process involving thorough washing with distilled water to eliminate any potential surface contaminants. Subsequently, the samples were subjected to a controlled drying process within an oven set at a precise temperature of 65 °C until reaching a constant weight, effectively removing moisture content. The grinded 1 g samples were taken, and 10 mL of HNO_3_ was added to it for digestion, kept overnight, and then added 4 mL of perchloric acid to it and heated on hot plates until the solution became transparent. Collect the samples in a 50 mL bottle and make the volume with distilled water. And then followed the procedure for the determination of P through a spectrophotometer [37]. For total nitrogen, the plant sample of 0.2 g was taken with a 1.1 g digestion mixture in the digestion tube, to which 3 mL of sulphuric acid was added and kept in the digestion chamber for 11–12 hr. to digest. After digestion, the sample was taken to the Kjeldhal’s for determination of nitrogen followed [38]. This formula was used to determine the amount of nitrogen.

$$\textrm{N}\left(\%\right)=\frac{\left(\textrm{Treat}.-\textrm{Blank}\right)\times 0.005\times 0.014\times 100}{\textrm{W}.\textrm{t}\ \textrm{of}\ \textrm{sample}\ \left(\textrm{g}\right)\times \textrm{vol}.\;\textrm{made}\ \left(\textrm{mL}\right)}$$

$$\textrm{N}\ \textrm{uptake}\ \left(\textrm{kg}\ {\textrm{ha}}^{-1}\right)=\frac{\textrm{Plant}\ \textrm{N}\ \textrm{conc}.\times \textrm{Yield}\ \left(\textrm{kg}\ \textrm{ha}^{-1}\right)}{100}$$

NUE was recorded using the formula given below:

$$\textrm{NUE}\ \left(\%\right)=\frac{\textrm{N}\ \textrm{uptake}\ \left(\textrm{kg}\ \textrm{ha}^{-1}\right)\ \textrm{from}\ \textrm{treated}\ \textrm{plots}-\textrm{N}\ \textrm{uptake}\ \left(\textrm{kg}\ \textrm{ha}^{-1}\right)\ \textrm{in}\ \textrm{control}\ \textrm{plot}\ }{\textrm{applied}\ \textrm{rate}\ \textrm{of}\ \textrm{N}\ \left(\textrm{kg}\ \textrm{ha}^{-1}\right)}$$

### Crop harvesting and yield measurement

Mature maize plants were harvested on October 11, 2020. Various agronomic parameters were recorded, including plant height, number of leaves per plant, number of grains per ear, 100-grain weight, biomass yield, grain yield, and stover yield. Plant height (cm) was measured with measuring tape by selecting ten plants randomly in each treatment. The numbers of grains ear^−1^ were recorded by randomly picking ten spikes from each treatment. The selected ears of each treatment were separately threshed, and grains were counted using an electronic grain counter and then averaged. Plants were weighed to record fresh biomass, and these plants, used for fresh biomass, were air-dried under ambient sunlight for 1 week to find the dry biomass of the plants.

Each plot at maturity was harvested and threshed for grain yield of maize to obtain grain yield. Using the procedure below, fresh and dry biomass values were represented as kilograms per hectare.

$$\textrm{Biological}\ \textrm{yield}\ \left(\textrm{kg}\ {\textrm{ha}}^{-1}\right)=\frac{\textrm{Biological}\ \textrm{yield}\ \textrm{of}\ \textrm{the}\ \textrm{whole}\ \textrm{plot}}{\textrm{Area}\ \textrm{of}\ \textrm{Plot}}\times 10000$$

$$\textrm{Grain}\ \textrm{Yield}\ \left(\textrm{kg}\ {\textrm{ha}}^{-1}\right)=\textrm{Biological}\ \textrm{Yield}-\textrm{Stover}\ \textrm{Yield}$$

$$\textrm{Stover}\ \textrm{Yield}\ \left(\textrm{kg}\ {\textrm{ha}}^{-1}\right)=\textrm{Biological}\ \textrm{Yield}-\textrm{Grain}\ \textrm{Yield}$$

Following threshing, 100 grains from each plot were counted and weighed using an electronic balance to get the weight data for 100 grains.

### Statistical analysis

All the data were statistically analyzed using [39] procedures. Analysis of variance (ANOVA) was calculated to compare fertilizer treatments with respect to various measured parameters. When significant effects of treatments were found, adjusted LSD values of Turkey’s test were calculated to compare the different fertilizer treatments. Minitab (version 12) and OriginPro were used to perform statistical analyses [40].

### Plant material collection and use permission

No permission is required for plant material. Seeds were purchased from the local market.

### Ethics approval and consent to participate

We all declare that manuscript reporting studies do not involve any human participants, human data, or human tissue. So, it is not applicable.

### Complies with international, national and/or institutional guidelines

This study complies with relevant institutional, national, and international guidelines.

## Results

### ^Soil^ NH_4_^+^ − N and NO_3_^−^-N concentrations

**Fig. 2:**
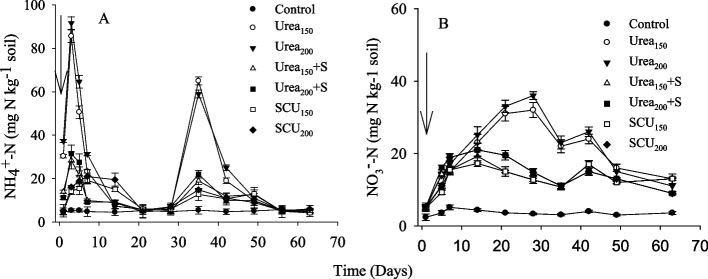
Ammonium (**a**) and nitrate (**b**) concentrations as affected by application of urea coated with S and S applied from gypsum. Values are means with standard error shown by vertical bars (*n* = 3). The solid arrows indicate the timing of N fertilization

The NH_4_^+^-N content was gradually altered during the experiment, as indicated in (Fig. 2a). The NH_4_^+^-N levels in soil increased considerably (*P* < 0.05), different between sulfur-coated urea and uncoated urea. In the first 7 days, soil NH_4_^+^-N was highest in urea_150_ and urea_200_, followed by urea_150_ + S and urea_200_ + S, while lowest in SCU_150_ and SCU_200_. Significantly more net NH_4_^+^-N was present in the soil for 63 days (327 mg kg^−1^) in urea_200_ followed by urea_150_ (314 mg kg^−1^). In SCU_200_ plot, net NH_4_^+^-N was 107 mg kg^−1^ followed by SCU_150_ 103 mg kg^−1^. Soil NO_3_^−^ -N concentration was also statistically (*P* < 0.05) different by the supplementation of sulfur-coated urea or with urea+S compared to urea alone (Fig. 2b). Temporal variations in soil NO_3_^−^ -N during the 63 experimental period showed that NO_3_^−^ -N concentration peaked on day 14 and started declining. Overall, NO_3_^−^ -N in soil was significantly (*P* < 0.05) higher than 102 and 113 mg kg^−1^ in urea_200_ followed by urea_150_. The total NO_3_^−^ -N in soil was less in SCU_150_ and SCU_200_ 35–44 mg kg^−1^, respectively.

### Ammonia volatilization

**Fig. 3:**
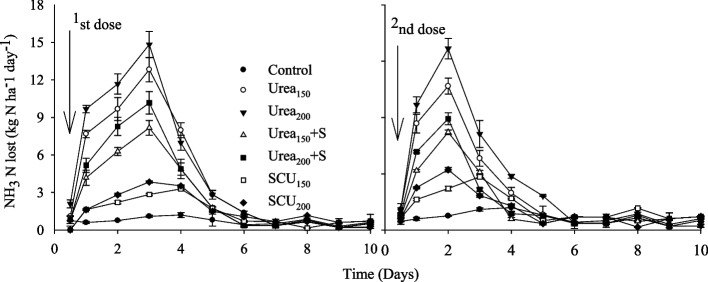
Fluxes of NH_3_ as affected by application of urea coated with S and S applied from gypsum. Values are means with standard error shown by vertical bars (*n* = 3). The solid arrows indicate the timing of N fertilization. Urea applied with 1st irrigation (1st dose) and urea applied at knee high stage (2nd dose)

Daily NH_3_ flux showed NH_3_ peaks after each urea fertilizer application to soils (Fig. 3). Over the sampling period, the highest NH_3_ emission peaks were observed 5 days after the urea application. After treatment application, the losses of NH_3_ increased rapidly, especially in urea-alone treatments compared to S-coated urea or with urea+S. As expected, urea_150_ and urea_200_ showed the highest percentage of NH_3_ losses, 80–85%, during the first week of its application. Nitrogen lost as NH_3_ of the applied N was higher (30–39%) in the urea_150_ and urea_200_ treatments (Table 2). Similarly, S-coated urea or with urea+S decreased N loss by 9–17%. Overall, cumulative NH_3_ losses were reduced (74–42%) when the S-coated urea or combined use of urea+S was applied compared with urea_200_ alone (Table 2).

**Table 2:** Effects S-coated urea on total ammonia losses (kg ha^−1^), N lost as NH_3_ (% of the applied N) and % difference in NH_3_ loss relative to urea at 200 kg N ha^−1^

Treatments	NH_**3**_emission (kg ha^**−1**^)	N lost as NH_**3**_ (% of the applied N)	% difference in NH_**3**_ loss relative to urea at 200 kg N ha^**−1**^
Control	1.6 ± 0.41^g^		
Urea_150_	47.8 ± 2.81^f^	30.8	
Urea_200_	61.3 ± 2.74^e^	39.8	
Urea_150_ + S	28.5 ± 1.51^d^	17.9	−58
Urea_200_ + S	35.6 ± 1.48^c^	17	−42
SCU_150_	15.8 ± 1.32^b^	09.1	−74
SCU_200_	21.2 ± 1.41^a^	09.8	−65

Within columns, means with the same letters are not significantly different at the *P < 0.05* level where *n =* 3*.* Means±standard errors (*n* = 3)

### Maize yield and yield attributes

**Fig. 4:**
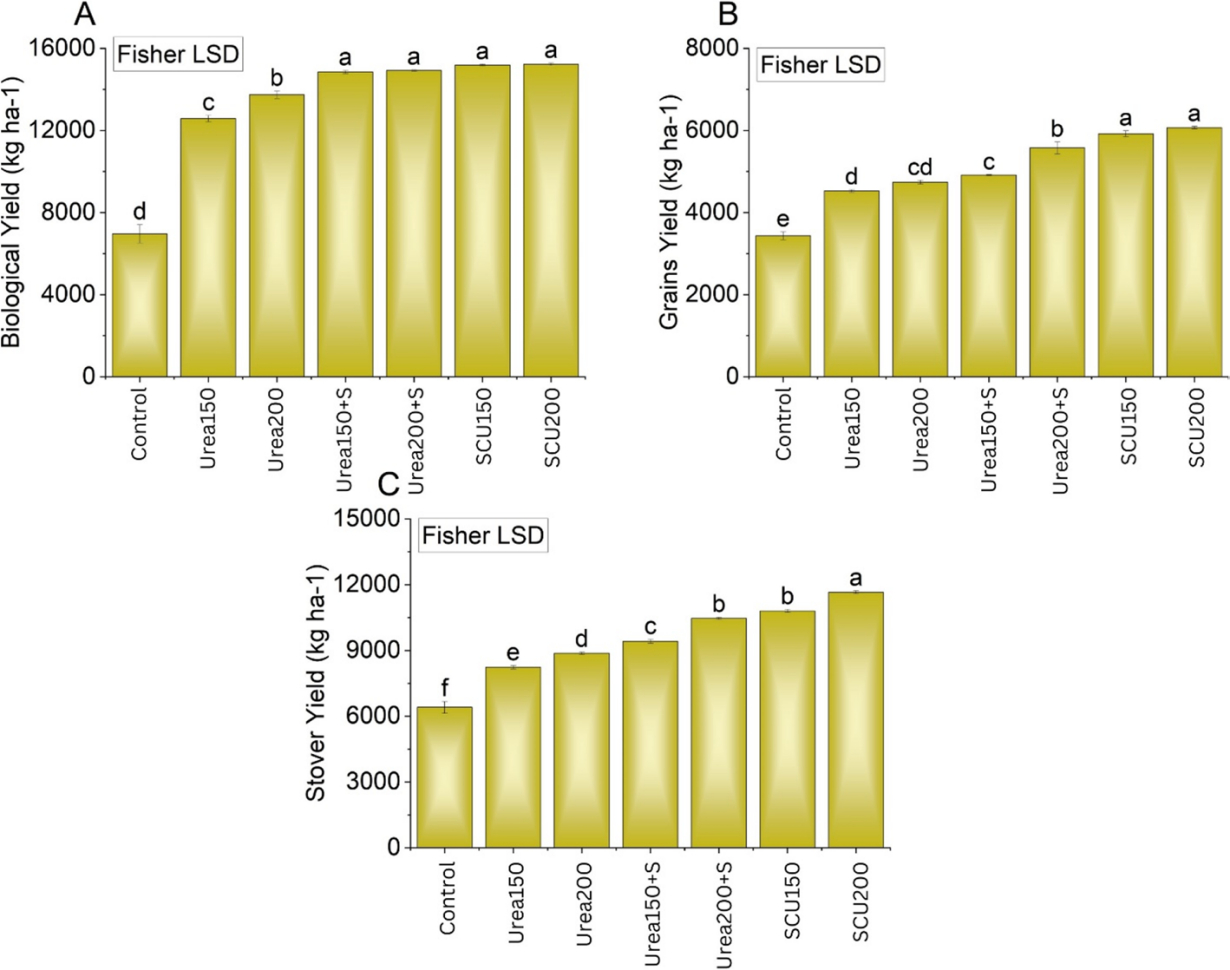
Effects of applying S from gypsum and S-coated urea on biological yield, grains yield and on stover yield. Bars are means of three replicated ± SE. Variable letters on bars show significant changes (*p* ≤ 0.05; Fisher LSD)

**Fig. 5:**
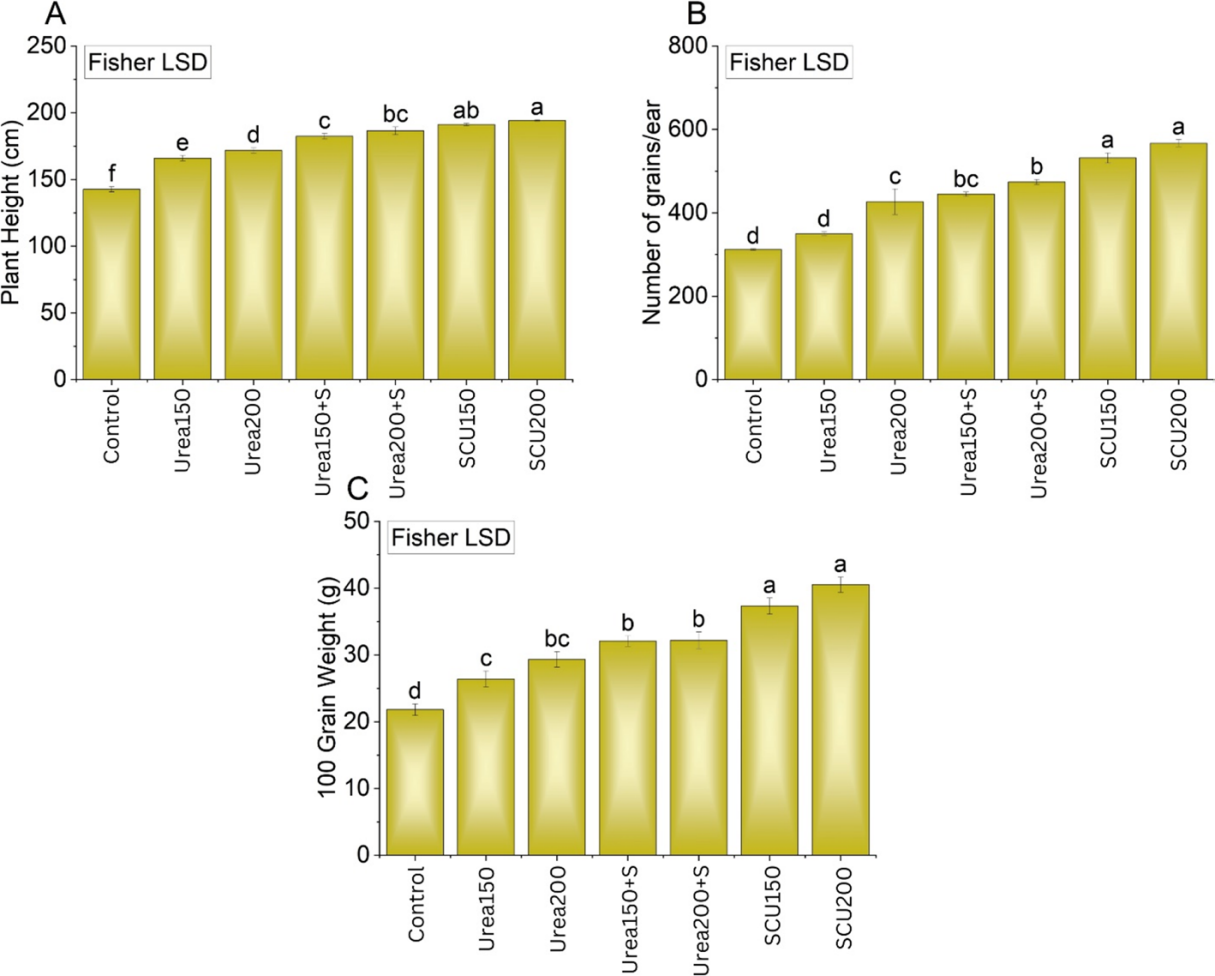
Effects of applying S from gypsum and S-coated urea on plant height, 100 grains weight and number of grains/ear. Bars are means of three replicated ± SE. Variable letters on bars show significant changes (p ≤ 0.05; Fisher LSD)

A significant (*P* < 0.05) change in the biological yield of maize was noted when different treatments were applied (Fig. 4a). Compared to urea_200_ alone, a better response from both urea+S and SCU was obtained. Combined application of urea_150_ + S and urea_200_ + S enhanced the biological yield of maize by 5.2 and 14%, respectively, compared with urea_200_ alone treatment. This increase in biological yield was much better, 22 and 30%, respectively, in SCU_150_ and SCU_200_ treatments compared with urea_200_ alone treatment. No significant change was noted among urea+SCU_150_ and SCU_200_ for biological yield. A significant (*P* < 0.05) change in the grain yield of maize was also noted when different treatments were applied (Fig. 4b). Maize grain yield was enhanced (4 and 17%) respectively when urea was applied in combination with urea_150_ + S and urea_200_ + S relative to the treatment with urea_200_ alone. The grain yield increased by 25 and 28% in the treatments SCU_150_ and SCU_200_ compared to the treatment with urea_200_ alone. No significant change was noted among SCU_150_ and SCU_200_ for grain yield. The application of treatments remained significantly (*P* < 0.05) different for stover yield (Fig. 4c). It was observed that both SCU_150_ and SCU_200_ performed significantly *P* < 0.05) better compared to all treatments for the enhancement in the stover yield. A significant variation was also noted where urea_150_ + S and urea_200_ + S were applied over urea_200_ alone for stover yield. Maximum enhancement of 21 and 32% in stover yield was noted where SCU_150_ and SCU_200_ were added compared to urea_200_ alone. However, both SCU_150_ and SCU_200_ did not differ from each other for alteration in stover yield. Plant height, grains ear^−1^, and 100-grain weight were also significantly (*P* < 0.05) influenced by the treatment application compared with control (Fig. 5a, b, and c). Both SCU_150_ and SCU_200_ significantly (P < 0.05) enhanced Plant height, grains ear^−1^, and 100-grain weight of maize compared with other treatments.

### Maize total N uptake and N response efficiency

**Fig. 6:**
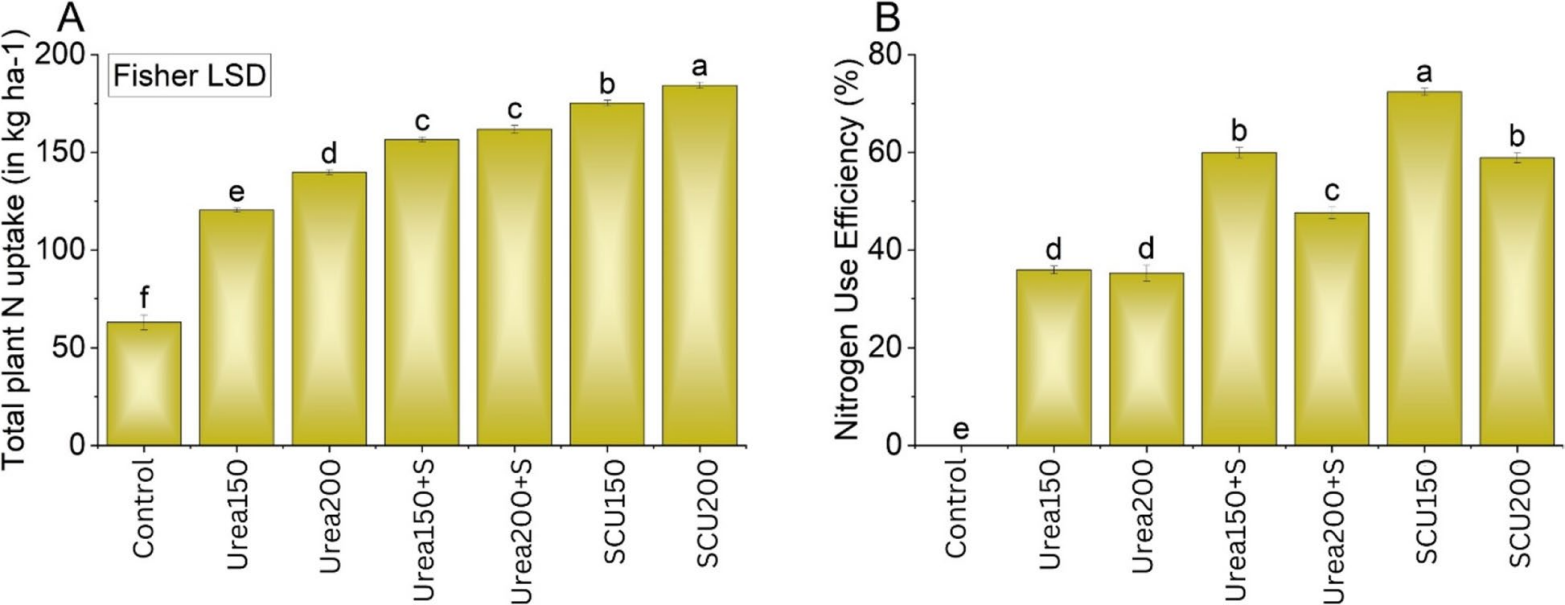
Effects of applying S from gypsum and S-coated urea on total plant N uptake and N use efficiency. Bars are means of three replicated ± SE. Variable letters on bars show significant changes (*p* ≤ 0.05; Fisher LSD)

The effect of applied treatments was significant (P < 0.05) for improving total N uptake in the plants (Fig. 6a). Results showed that both SCU_150_ and SCU_200_ treatments gave the highest total N uptake compared to urea_200_ alone. Maximum enhancement (26 and 31%) in total plant N uptake was noted where SCU_150_ and SCU_200_ were added as treatment compared to urea_200_ alone. Similarly, N use efficiency was also significantly (P < 0.05) greater in the treatments with urea in combination, i.e., urea+S or SCU (Fig. 6b). The N use efficiency values were 34, 36, 62, 52, 72, and 62% in the treatments with, urea_150_ alone, urea_200_ alone, urea_150_ + S, urea_200_ + S, SCU_150_ and SCU_200_, respectively.

### Convex hull and hierarchical cluster analysis

**Fig. 7:**
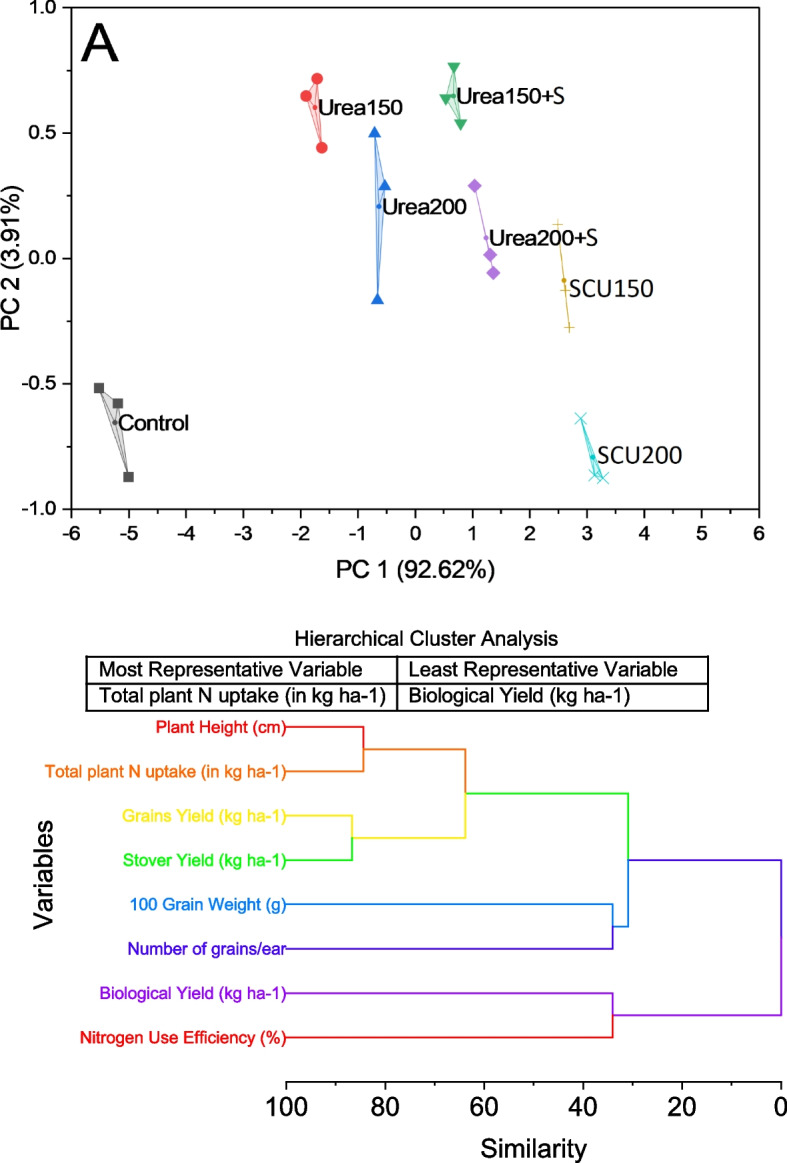
Convex hull for treatments and hierarchical cluster plot for studied attributes

In the presented convex hull cluster plot, we explore the distribution and clustering of data points derived from a dataset characterized by principal components (PC) 1 and 2, accounting for 92.62 and 3.91% of the total variance, respectively. The dataset encompasses various experimental treatments, namely control, urea_150_, urea_200_, urea_150_ + S, urea_200_ + S, SCU_150_, and SCU_200_, each associated with specific sets of PC1 and PC2 scores. The plot elegantly visualizes the clustering patterns of these treatments in the two-dimensional space defined by PC1 and PC2. The presented plot shows that the treatment labeled urea_150_ + S occupies a distinct position in the two-dimensional space defined by PC1 and PC2. This treatment demonstrates a clear separation from the other clusters, indicating a unique profile regarding the PC1 and PC2 scores (Fig. 7a).

Two prominent clusters are observed in the dendrogram. The first cluster includes grains yield (kg ha^−1^) and stover yield (kg ha^−1^), which exhibit a high level of similarity with a dissimilarity measure of 13.29071. This suggests that these two variables share common patterns or responses in the dataset, possibly indicating a strong association between grain and stover yields. The second cluster comprises four variables: plant height (cm), total plant n uptake (in kg ha^−1^), biological yield (kg ha^−1^), and nitrogen use efficiency (%). These variables are grouped due to their relatively lower dissimilarity measures, with plant height and total plant n uptake having a dissimilarity of 15.57105, and biological yield and nitrogen use efficiency having a dissimilarity of 65.94193. This clustering implies that these agricultural traits may exhibit similar trends or responses in the dataset. Furthermore, a third cluster consists of 100-grain weight (g) and the number of grains/ear), closely related with a dissimilarity measure of 65.95871. These variables may be indicative of traits related to grain size and production on a per-ear basis. Finally, two variables, 11 and 12, do not cluster with any other variables in the analysis, indicating that they exhibit dissimilar patterns compared to the rest of the traits. These two variables’ specific nature and significance would require further investigation and context from the original data.

### Effects of urea coated with sulfur and sulfur applied from gypsum on NH_3_ emissions

In our experiment, we observed a fast increase in NH_4_^+^ content at first 2–3 days after application of urea (Fig. 2), which could be ascribed to quick hydrolysis of urea as a result more NH_4_^+^ (Fig. 2) and OH^−^ ions are produced [41] and allowing significant NH_3_ losses (Fig. 3). We observed 65–74% reduction in NH_3_ emission with SCU compared to urea_200_ alone treatment (Table 2). This reduction could slow urea hydrolysis because S-coated urea acts as a urease inhibitor, which adversely affects the activities of soil enzymes that could reduce the hydrolysis activities and reduce the loss of NH_3_ from soil [42–44]. Slow urea hydrolysis reduced the release of NH_4_^+^ (Fig. 2 a) and minimized the possibility of a sudden rise in pH that occurs during urea hydrolysis [43, 45, 46], resulting in a reduction of NH_3_ emissions (Fig. 3; Table 2). The amendments of S reduce the pH of the rhizosphere, which linearly controls the nitrification process and reduces the loss of N from soil [12]. It has also been reported that S has low solubility in water, retains the NH_3_ in the soil for a longer period [47, 48], and prolongs the transformation of urea to NH_4_^+^ and NO_3_^−^ (Fig. 2). Furthermore, slow urea hydrolysis caused by S treatments may also allow more time for irrigation or rainfall to transfer the applied urea to the subsoil layers vertically and laterally, protecting the applied N from losses [7, 49, 50].

### Effects of urea coated with sulfur and sulfur applied from gypsum on maize yield, N uptake and N use efficiency

Sulfur-coated urea significantly increased maize yield, yield components total N uptake compared to the treatment with urea alone. This increase could be due to the slow release of N from urea (Fig. 2) and, therefore, reduced N losses as NH_3_ (Table 2). Shivay et al. [27] suggested that S-coated urea as a source of N and S may have raised N and S concentrations in spring wheat, boosting plant uptake and enhancing crop output and yield-related characteristics [51]. The slow release of NH_4_^+^ rather than NO_3_^−^ for several days after urea coated with S likely contributed to these increases as well, giving plants more time to absorb N in the form of NH_4_^+^, which can then be incorporated into organic compounds and eventually plant protein at a lower energy cost than NO_3_^−^ [52]. Slow release of NH_4_^+^ owing to S improves the environment by limiting NH_3_ emission (Table 2). However, it also benefits agriculture and the economy by improving the efficiency with which N is used (Fig. 6) [27, 53], especially in N-deficient soils.

The increase of yield and yield components by S application could be that S in the plant body synthesizes sulfur-containing amino acids, the major component of proteins [52]. Sulfur application could also increase the absorption of N, due to which the content of protein and the proportions of glutenin and gliadin in total protein affect the growth and quality of the crop (Tao et al. 2018). Sulfur fertilization could also increase S-bearing protein Met’s content and thereby improve cereal yield and nutritional quality [54]. Higher uptake of N in S-coated urea treatments resulted in enhanced Plant height, grains ear^−1^, and 100-grain weight (Fig. 5), which could reduce N losses [55, 56], thus improving plant availability of N. urea coating with nutrients also reduces their deficiencies and contribute to yield enhancement in cereals [57]. Urea is a highly volatile compound, so excessive N is lost to the environment and becomes unavailable for plants. The major contribution of coated urea in enhancing NUE is the slow release of N from fertilizers [56]. Accessibility of more N to plants improved nitrogen recovery, chlorophyll, and total dry matter [55].

Overall, urea coated with S resulted in significantly improved N use efficiency relative to the urea alone treatment, especially at the low N application rate (Fig. 6b). Our findings concur with those of [27, 58], who also noted that the application of S linearly increases the NUE by 50% in crops. The supplementations of S-coated urea delay the hydrolysis of urea and enhance the mobility of N in soil, which plays an active role in the plant’s physiochemical properties (Li et al., 2018; Zaman et al., 2013). Additionally, S-coated urea has lower NH_3_ losses of N (Fig. 3, Table 2), which may promote plant N recovery [59, 60]. The combined application of S and N may increase the concentration of S in a grain of maize crops to enhance their nutritional value, which is likely to be a significant step toward improving human health.

## Conclusion

In conclusion, the utilization of S-coated urea at a rate of 150 kg N ha^−1^ could prove to be an efficient approach to reducing the necessity for excessive urea fertilizer application. This not only helps in mitigating the emission of ammonia (NH_3_) but also enhances nitrogen use efficiency (NUE) and ultimately leads to an increase in maize yield. It is advisable to conduct further investigations across various soil types and under different climatic conditions to solidify the recommendation of applying S-coated urea at a rate of 150 kg N ha^−1^ as the optimal method for promoting the growth and yield of maize.

## Data Availability

All data generated or analysed during this study are included in this published article.

## References

[1] Driver JG, Owen RE, Makanyire T, Lake JA, McGregor J, Styring P (2019). Blue urea: fertilizer with reduced environmental impact. Front Energy Res.

[2] Hube S, Salazar F, Rodríguez M, Mejías J, Ramírez L, Alfaro M (2022). Dynamics of nitrogen gaseous losses following the application of foliar Nanoformulations to grasslands. J Soil Sci Plant Nutr.

[3] Dawar K, Khan H, Zaman M, Muller C, Alam SS, Fahad S, et al. The effect of biochar and nitrogen inhibitor on ammonia and nitrous oxide emissions and wheat productivity. J Plant Growth Regul. 2021; 10.1007/s00344-020-10283-1.

[4] Shan L, He Y, Chen J, Huang Q, Wang H (2015). Ammonia volatilization from a Chinese cabbage field under different nitrogen treatments in the Taihu Lake Basin, China. J Environ Sci.

[5] Pelster DE, Chantigny MH, Angers DA, Bertrand N, MacDonald JD, Rochette P (2018). Can soil clay content predict ammonia volatilization losses from subsurface-banded urea in eastern Canadian soils?. Can J Soil Sci.

[6] Sidi N, Aris AZ, Talib SN, Johan S, Yusoff TSTM, Ismail MZ (2015). Influential factors on the cation exchange capacity in sediment of Merambong shoal, Johor. Procedia Environ Sci.

[7] Dawar K, Zaman M, Rowarth JS, Blennerhassett J, Turnbull MH (2011). Urease inhibitor reduces N losses and improves plant-bioavailability of urea applied in fine particle and granular forms under field conditions. Agric Ecosyst Environ.

[8] Jones CA, Koenig RT, Ellsworth JW, Brown BD, Jackson GD (2007). Management of urea fertilizer to minimize volatilization.

[9] Proctor C, Koenig R, Johnston W (2010). Potential for ammonia volatilization from urea in dryland Kentucky bluegrass seed production systems. Commun Soil Sci Plant Anal.

[10] Afshar RK, Lin R, Mohammed YA, Chen C (2018). Agronomic effects of urease and nitrification inhibitors on ammonia volatilization and nitrogen utilization in a dryland farming system: field and laboratory investigation. J Clean Prod.

[11] Cameron KC, Di HJ, Moir JL (2013). Nitrogen losses from the soil/plant system: a review. Ann Appl Biol.

[12] Beig B, Niazi MBK, Jahan Z, Kakar SJ, Shah GA, Shahid M (2020). Biodegradable polymer coated granular urea slows down n release kinetics and improves spinach productivity. Polymers (Basel).

[13] Klimczyk M, Siczek A, Schimmelpfennig L (2021). Improving the efficiency of urea-based fertilization leading to reduction in ammonia emission. Sci Total Environ.

[14] Dimkpa CO, Fugice J, Singh U, Lewis TD (2020). Development of fertilizers for enhanced nitrogen use efficiency--Trends and perspectives. Sci Total Environ.

[15] Snyder CS (2017). Enhanced nitrogen fertiliser technologies support the ‘4R’ concept to optimise crop production and minimise environmental losses. Soil Res.

[16] Lawrencia D, Wong SK, Low DYS, Goh BH, Goh JK, Ruktanonchai UR (2021). Controlled release fertilizers: A review on coating materials and mechanism of release. Plants..

[17] Andrade AB, Guelfi DR, Chagas WFT, Cancellier EL, de Souza TL, Oliveira LSS (2021). Fertilizing maize croppings with blends of slow/controlled-release and conventional nitrogen fertilizers. J Plant Nutr Soil Sci.

[18] Santos CF, da Silva Aragao OO, Silva DRG, da Conceição JE, Chagas WFT, Correia PS (2020). Environmentally friendly urea produced from the association of N-(n-butyl) thiophosphoric triamide with biodegradable polymer coating obtained from a soybean processing byproduct. J Clean Prod.

[19] Apostolopoulou E. The global market for slow-release, controlled-release and stabilized fertilizers. Beijing Int Fertil Assoc. 2016;

[20] Guelfi D (2017). Fertilizantes nitrogenados estabilizados, de liberação lenta ou controlada. Informações Agronômicas.

[21] Ag P-C, Chinchilla-Soto C, Elizondo-Salazar JA, Barboza R, Kim D-G, Müller C (2021). Nitrification inhibitor nitrapyrin does not affect yield-scaled nitrous oxide emissions in a tropical grassland. Pedosphere.

[22] Monge-Munoz M, Urquiaga S, Müller C, Cambronero-Heinrichs JC, Zaman M, Chinchilla-Soto C (2021). Nitrapyrin effectiveness in reducing nitrous oxide emissions decreases at low doses of urea in an andosol. Pedosphere..

[23] Wilson TL, Guttieri MJ, Nelson NO, Fritz A, Tilley M (2020). Nitrogen and sulfur effects on hard winter wheat quality and asparagine concentration. J Cereal Sci.

[24] Li N, Yang Y, Wang L, Zhou C, Jing J, Sun X (2019). Combined effects of nitrogen and sulfur fertilization on maize growth, physiological traits, N and S uptake, and their diagnosis. F Crop Res.

[25] Shivay YS, Pooniya V, Pal M, Ghasal PC, Bana R, Jat SL (2019). Coated urea materials for improving yields, profitability, and nutrient use efficiencies of aromatic rice. Glob Challenges.

[26] Xin Y, Wenhai M, Shaofu W, Lianghuan W, Jianqiu C (2017). Rice responses to single application of coated urea on yield, dry matter accumulation, and nitrogen uptake in southern China. J Plant Nutr.

[27] Shivay YS, Pooniya V, Prasad R, Pal M, Bansal R (2016). Sulphur-coated urea as a source of Sulphur and an enhanced efficiency of nitrogen fertilizer for spring wheat. Cereal Res Commun.

[28] Patel S, Goyal A (2015). Applications of natural polymer gum Arabic: A review. Int J Food Prop.

[29] Shivay YS, Prasad R, Pal M (2016). Effect of nitrogen levels and coated urea on growth, yields and nitrogen use efficiency in aromatic rice. J Plant Nutr.

[30] Araújo ED, Marsola T, Miyazawa M, Soares LH, Urquiaga S, Boddey RM, Alves BJ (2009). Calibration of a semi-opened static chamber for the quantification of volatilized ammonia from soil. Pesqui Agropecu {á} ria Bras.

[31] Jantalia CP, Halvorson AD, Follett RF, Rodrigues Alves BJ, Polidoro JC, Urquiaga S (2012). Nitrogen source effects on ammonia volatilization as measured with semi-static chambers. Agron J.

[32] Keeney DR, Nelson DW, Page AL (1983). Nitrogen-inorganic forms. Methods of soil analysis: part 2 chemical and microbiological properties, 9.2.2. 2nd edition.

[33] Page AL, Miller RH, Keeny DR (1982). Soil pH and lime requirement. Methods of soil analysis. 2nd edition.

[34] Rhoades JD (1993). Electrical conductivity methods for measuring and mapping soil salinity. Adv Agron.

[35] Nelson DW, Sommers LE, Page AL (1982). Total carbon, organic carbon, and organic matter. Methods of soil analysis: part 2 chemical and microbiological properties.

[36] Gee GW, Bauder JW, Klute A (1986). Particle-size analysis. Methods of soil analysis: part 1—physical and mineralogical methods.

[37] Bardsley CE, Lancaster JD (1960). Determination of reserve sulfur and soluble sulfates in soils. Soil Sci Soc Am J.

[38] Bremner JM, Mulvaney CS, Page AL, Miller RH, Keeney DR (1982). Nitrogen–total. Methods of soil analysis. Part 2. Chemical and microbiological properties.

[39] Steel RG, Torrie JH, Dickey DA (1997). Principles and procedures of statistics: A biometrical approach.

[40] OriginLab Corporation (2021). OriginPro.

[41] Sanz-Cobena A, Lassaletta L, Estellés F, Del Prado A, Guardia G, Abalos D (2014). Yield-scaled mitigation of ammonia emission from N fertilization: the Spanish case. Environ Res Lett.

[42] Hopkins BG (2020). Developments in the use of fertilizers. Achieving sustainable crop nutrition.

[43] Trenkel ME (2021). Slow- and controlled-release and stabilizedfertilizers: an option for enhancing nutrient use Efficiencyin agriculture.

[44] Bolan N, Saggar S, Singh J (2004). The role of inhibitors in mitigating nitrogen losses in grazed pasture.

[45] Cantarella H, Otto R, Soares JR, de Brito Silva AG (2018). Agronomic efficiency of NBPT as a urease inhibitor: a review. J Adv Res.

[46] Dawar K, Zaman M, Rowarth JS, Blennerhassett J, Turnbull MH (2011). Urea hydrolysis and lateral and vertical movement in the soil: effects of urease inhibitor and irrigation. Biol Fertil Soils.

[47] Rahman NS, Yunus R, Ishak CF, Hanif KS (2018). Laboratory evaluation on ammonia volatilization from coated urea fertilizers. Commun Soil Sci Plant Anal.

[48] Vashishtha M, Dongara P, Singh D (2010). Improvement in properties of urea by phosphogypsum coating. Int J ChemTech Res.

[49] Shujrah AA, Mohd KY, Hussin A, Othman R, Haruna O (2010). Impact of potassium humate on selected chemical properties of an Acidic soil. 19th World Congress of Soil Science.

[50] Khariri RBA, Yusop MK, Musa MH, Hussin A (2016). Laboratory evaluation of metal elements urease inhibitor and DMPP nitrification inhibitor on nitrogenous gas losses in selected Rice soils. Water Air Soil Pollut.

[51] Ghafoor I, Habib-ur-Rahman M, Ali M, Afzal M, Ahmed W, Gaiser T (2021). Slow-release nitrogen fertilizers enhance growth, yield, NUE in wheat crop and reduce nitrogen losses under an arid environment. Environ Sci Pollut Res.

[52] Aarabi F, Naake T, Fernie AR, Hoefgen R (2020). Coordinating sulfur pools under sulfate deprivation. Trends Plant Sci.

[53] Ke J, He R, Hou P, Ding C, Ding Y, Wang S (2018). Combined controlled-released nitrogen fertilizers and deep placement effects of N leaching, rice yield and N recovery in machine-transplanted rice. Agric Ecosyst Environ.

[54] Klikocka H, Cybulska M, Barczak B, Narolski B, Szostak B, Kobiałka A (2016). The effect of Sulphur and nitrogen fertilization on grain yield and technological quality of spring wheat. Plant Soil Environ.

[55] Wang HH, Hegazy AM, Jiang X, Hu ZY, Lu J, Mu J (2016). Suppression of ammonia volatilization from rice–wheat rotation fields amended with controlled-release urea and urea. Agron J.

[56] Li P, Lu J, Wang Y, Wang S, Hussain S, Ren T (2018). Nitrogen losses, use efficiency, and productivity of early rice under controlled-release urea. Agric Ecosyst Environ.

[57] Noor Affendi NM, Yusop MK, Othman R (2018). Efficiency of coated urea on nutrient uptake and maize production. Commun Soil Sci Plant Anal.

[58] Zareabyaneh H, Bayatvarkeshi M (2015). Effects of slow-release fertilizers on nitrate leaching, its distribution in soil profile, N-use efficiency, and yield in potato crop. Environ Earth Sci.

[59] Wei X, Chen J, Gao B, Wang Z, Srivastava AK, Chengxiao H (2020). Role of controlled and slow release fertilizers in fruit crop nutrition. Fruit Crops.

[60] Mustafa A, Athar F, Khan I, Chattha MU, Nawaz M, Shah AN (2022). Improving crop productivity and nitrogen use efficiency using sulfur and zinc-coated urea: A review. Front Plant Sci.

